# Mutagenesis of Snu114 domain IV identifies a developmental role in meiotic splicing

**DOI:** 10.1080/15476286.2018.1561145

**Published:** 2019-01-23

**Authors:** Amit Gautam, Jean D. Beggs

**Affiliations:** Wellcome Centre for Cell Biology, University of Edinburgh, Edinburgh, UK

**Keywords:** Snu114/regulated splicing/Cwc21/meiosis/PP1/U5 snRNP

## Abstract

Snu114, a component of the U5 snRNP, plays a key role in activation of the spliceosome. It controls the action of Brr2, an RNA-stimulated ATPase/RNA helicase that disrupts U4/U6 snRNA base-pairing prior to formation of the spliceosome’s catalytic centre. Snu114 has a highly conserved domain structure that resembles that of the GTPase EF-2/EF-G in the ribosome. It has been suggested that the regulatory function of Snu114 in activation of the spliceosome is mediated by its C-terminal region, however, there has been only limited characterisation of the interactions of the C-terminal domains. We show a direct interaction between protein phosphatase PP1 and Snu114 domain ‘IVa’ and identify sequence ‘YGVQYK’ as a PP1 binding motif. Interestingly, this motif is also required for Cwc21 binding. We provide evidence for mutually exclusive interaction of Cwc21 and PP1 with Snu114 and show that the affinity of Cwc21 and PP1 for Snu114 is influenced by the different nucleotide-bound states of Snu114. Moreover, we identify a novel mutation in domain IVa that, while not affecting vegetative growth of yeast cells, causes a defect in splicing transcripts of the meiotic genes, *SPO22, AMA1* and *MER2*, thereby inhibiting an early stage of meiosis.

## Introduction

Most eukaryotic precursors of messenger RNAs (pre-mRNAs) contain introns that disrupt the protein coding sequences. Introns are removed by a large protein-RNA complex called the spliceosome. Spliceosomes consist of around a hundred [1] (depending on the organism) proteins and five small nuclear ribonucleoprotein particles (snRNPs) (U1, U2, U4, U5 and U6) that assemble in an orderly manner on the pre-mRNA. The U1 snRNP interacts with the 5ʹ end of the intron via a base-pairing interaction between a short conserved sequence at the 5ʹ splice site (5’SS) and a complementary sequence near the 5ʹ end of U1 small nuclear RNA (snRNA). The U2 snRNA in the U2 snRNP base pairs with the branch site sequence in the intron to form the pre-spliceosome, or A complex. Addition of a pre-assembled U4/U6•U5 triple snRNP produces B complex. The U4 and U6 snRNAs share extensive sequence complementarity and are base-paired as a heterodimer within the U4/U6**·**U5 tri-snRNP complex. The B complex undergoes a series of ATP-dependent structural rearrangements that allow the U1 and U4 snRNPs to dissociate and the NineTeen Complex (NTC in yeast; Prp19-CDC5 complex in humans) proteins are recruited, leading to formation of the B^act^ complex that when activated forms the B* complex. C complex is generated upon cleavage of the 5’SS in the first catalytic step. The RNA products of this reaction, 5ʹexon and lariat intron-3ʹexon, are repositioned as substrates for second step catalysis, in which the 3’splice site (3’SS) is cleaved and, simultaneously, the exons are joined to form the spliced mRNA [1,2].

Snu114 is a core component of the U5 snRNP that is present in the spliceosome throughout the splicing cycle. Snu114 is the only GTP-binding protein involved in splicing and has a highly conserved five-domain structure that is strikingly homologous to the ribosomal elongation factor EF-2 (EF-G in prokaryotes). Using a random mutagenesis approach, Brenner and Guthrie [3] identified conditionally lethal alleles of *SNU114* that blocked the first step of splicing *in vivo* and *in vitro*. The allele *snu114-60*, which is truncated at the C-terminus, caused cold-sensitive growth and was found to block U4 snRNP release and spliceosome activation [4]. This allele was synthetically lethal with mutations in *PRP8* and in factors required for activation of the spliceosome, including the DExD/H-box ATPases Brr2 and Prp28 [3]. It was suggested that GTP hydrolysis results in a rearrangement between Prp8 and the C-terminus of Snu114 that leads to release of U1 and U4 through the action of Prp28 and Brr2 [4]. In addition, Snu114 triggered U4/U6 unwinding when bound to GTP but not when bound to GDP [5]. The GTP/GDP state similarly regulated spliceosome disassembly during recycling of spliceosome components for subsequent rounds of splicing [5]. However, it was subsequently reported that the G-domain of Snu114, although able to bind GTP, may lack GTPase activity [6].

Based on cryo-electron microscopy (cryo-EM) reconstruction of the U4/U6.U5 tri-snRNP from budding yeast it was proposed that Snu114 may bring the Brr2 helicase in close proximity with its substrate, the U4/U6 snRNA hetero-dimer, and may also play a role in positioning the U5 snRNA to insert its loop I for proper alignment of exons for catalysis [6]. According to the cryo-EM structure of the yeast spliceosome immediately after the first catalytic step the alignment of the 5ʹexon may involve Watson Crick base-paired interactions between the bases at 5’SS −2,-3,-4 (usually AAA [7]) and the U-rich loop 1 of U5 snRNA [8]. How might Snu114 perform these roles? On the basis of genetic interactions between *SNU114* and mutations that affect RNA interactions in the spliceosome, Frazer et al [9] hypothesized that the G-domain of Snu114 directly or indirectly senses RNA interactions in the spliceosome and, through its C-terminal domain, regulates other proteins, such as Brr2 and Prp28, that bring about RNA/RNA rearrangements required for splicing.

Grainger et al [10] demonstrated a physical interaction between Snu114, Prp8 and Cwc21, a 135-residue component of the yeast B^act^ complex and an ortholog of human alternative splicing factor SRm300/SRRM2 [10]. The conserved cwf21 domain of both Cwc21 and SRm300, binds directly with Prp8 in its so-called Snu114/Cwc21 interacting domain (SCwid). SRm300/SRRM2 associates strongly with the human spliceosomal C complex (in 1M NaCl) [11,12] and recent cryo-EM spliceosome structures show Cwc21/SRm300 in the catalytic centre [13,14]. Cwc21 also binds directly to the C-terminus of Snu114 [10], a region that (by analogy to EF-G and EF-2 in the ribosome) structurally mimics RNA [15–17]. Cwc21 has strong genetic links to Isy1, a component of the Nineteen Complex (NTC); simultaneous deletion of both *CWC21* and *ISY1* inhibits step 1 of splicing at elevated temperatures [10,18].

Yeast cells undergo meiosis and sporulation when starved for nitrogen and fermentable carbon source [19]. These events are regulated transcriptionally [20,21]. Ime1 acts as a master regulator of the sporulation process and its expression induces sporulation during vegetative growth in *MAT* insufficient strains [22,23]. The activation of Ime1 initiates the transcription of early meiotic genes [24]. In vegetative growth, these genes are repressed by Ume6 binding to the URS1 site found in the promoter region of early meiosis specific genes [25,26].

Most studies on regulated splicing have focused on the *MER1*-regulon. *MER1* encodes a splicing enhancer protein that is expressed early during meiosis [27,28]. Nam8, a splicing factor expressed both during vegetative growth and meiosis, is required for Mer1 function to control subsets of genes during meiosis [28,29]. The Mer1 protein binds directly to an enhancer sequence (‘AUACCCUU’) found in the regulated introns of target genes. In the absence of Mer1 and Nam8 the pre-mRNA transcripts corresponding to these genes are not spliced [29,30], inhibiting sporulation. So far, four intron-containing meiosis specific genes, *MER2, MER3/HFM1, SPO22* and *AMA1*, have been identified to contain this enhancer sequence and are therefore dependent upon *MER1* for splicing during meiosis. In addition to the enhancer sequence, these *MER1* dependent introns contain non-consensus 5’SS suggesting an additional role for Mer1 in stabilizing 5’SS and U1 snRNA interaction.

Splicing is regulated in part by protein phosphorylation and dephosphorylation [31], about which relatively little is known. The activities of two protein phosphatases, PP1 and PP2A, are essential for both steps of splicing [32,33]. PP1 contains a catalytic subunit, PP1c that is highly conserved in eukaryotes. Glc7, the PP1c in *Saccharomyces cerevisiae*, has 81% amino acid sequence identity with the rabbit PP1 (rPP1) catalytic subunit [34]. PP1c can associate with a spectrum of interacting subunits that confer substrate specificity [35]. We present evidence for interaction of PP1 and Cwc21 with a putative PP1 binding motif in Snu114 domain ‘IVa’. Mutational analysis of Snu114 domain IV (IVa and IVb) identified alleles that, by themselves, cause no growth defect, but display temperature-dependent growth defects when combined with *isy1Δ*. Intriguingly, one of these alleles in domain ‘IVa’ inhibits meiosis, apparently by causing a defect in splicing several meiotic transcripts. We propose a model in which Cwc21 and PP1 compete to modulate Snu114 function, with consequences for splicing fidelity that may be critical in meiosis.

## Results

### Mutants of SNU114 have genetic interactions with CWC21 and ISY1

**Figure 1.**
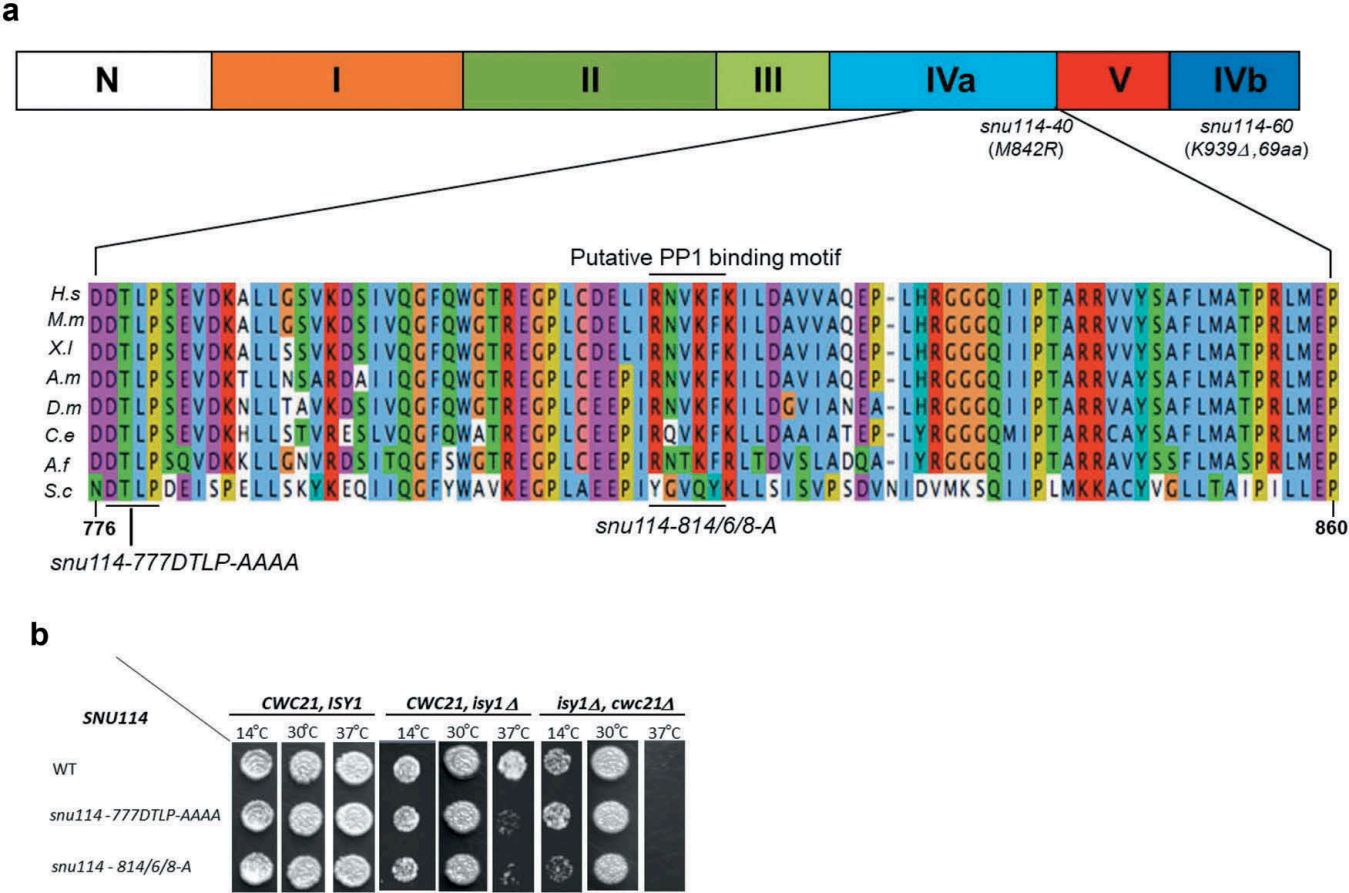
*SNU114 interacts genetically with CWC21 and ISY1*. (a) Snu114 contains five structural domains (I-V). The N-terminus contains a non-conserved acidic region. Domain I contains the consensus G-domain within which elements G1-G5 are important for binding GTP. The G-domain is present in Snu114 and EF-2 but not in bacterial EF-G proteins. Domain ‘IV’ is postulated to move 37Å upon GTP binding based on its homology to the two ribosomal GTPases, EF-G and EF-Tu (EF-2 in eukaryotes). The zoomed region of highly conserve domain ‘IVa’ shows an alignment of aa776-aa860 (*Saccharomyces cerevisiae* numbers) with the same region from various organisms: H.s, *Homo sapiens*; M.m, *Mus musculous*; X.l, *Xenopus laevis*; A.m, *Apis melligera*; D.m, *Drosophila melanogaster*; C.e, *Caenorhabditis elegans*; A.f, *Aspergillus fumigatus*; S.c, *Saccharomyces cerevisiae*. (b) Growth defects observed in plasmid shuffle assay. After shuffling out the wild-type plasmid, yeast cells expressing Snu114 from the mutated plasmids (indicated in the figure) were grown to stationary phase in YPDA medium. Then, cells diluted to OD_600_ of 0.3 were spotted on YPDA plates and grown at 30°C or 37°C for two days or at 14°C for eight days.

Domains IVa and IVb (Fig. 1A) of Snu114 are physically close in the modelled tertiary structure [3] and in the cryo-EM structure of the tri-snRNP [6] and, potentially might function as a structural unit. To further investigate the functions of these two domains, we performed alanine scanning mutagenesis. Twenty novel mutations were screened for growth defects (Supplementary Fig. S1 and Table T1), with none of these causing any discernible defect during vegetative growth, even at 37°C, at which temperature growth of the previously isolated *snu114-40* mutant (also in domain IVa) is inhibited [3,36] (Supplementary Fig. S1 and Table T1). The fact that alanine scanning mutagenesis of the C-terminus did not identify other individual residues that are essential for growth is perhaps not surprising, as the *snu114-60* mutant that has a large deletion in domain IVb has only a mild growth defect at low temperature [3], indicating a robust system.

In view of the known physical and genetic interactions of Cwc21 with domain IVa [10], we performed genetic tests (using plasmid shuffle strains; see Materials and Methods) between the new *snu114* alleles and *cwc21Δ* as well as *isy1Δ* that is synthetic lethal with *cwc21Δ* at 37ºC. Two alleles, *snu114-777DTLP-AAAA* (substitution with A at each of the four positions) and *snu114-814/6/8-A* (substituted with As at all three positions) were found to cause heat-sensitive growth at 37ºC when combined with *isy1∆* (Fig. 1B, Supplementary Fig. S1 and Table 1). Furthermore, deletion of both *ISY1* and *CWC21* caused cold-sensitive growth at 14ºC when combined with *snu114-814/6/8-A*, (Fig. 1B, Supplementary Fig. S1).

### ‘YGVQYK’ is a novel PP1 binding motif

The regulatory subunit and substrates of PP1 usually contain a conserved binding motif, with the consensus [RK]-x (0, 1)-V-x- F or F-x-x-(RK)-x-(RK), that binds to a small hydrophobic groove on the surface of PP1c [37]. Sequence alignment of Snu114 domain IVa from various species identified a putative PP1 binding motif ‘RxVxF’ (Fig. 1A), that corresponds to the sequence mutated in *snu114-814/6/8-A*.

We hypothesized that PP1 may interact here to regulate the activity of Snu114 and/or other interacting proteins. In addition, the putative yeast PP1 binding motif (Snu114-Y814-K819) and 777-DTLP-780 region are close to each other in the tri-snRNP structure (5GAM [6], Supplementary Fig. S2). A hydrogen bond is formed between Snu114-P780 and Snu114-K819 in the structure immediately after branching (5LJ3 [8], Supplementary Fig. S2) suggesting interaction.

We therefore tested whether yeast Snu114 interacts with the yeast PP1c protein Glc7 in a yeast two-hybrid (Y2H) assay. Full-length Snu114 fused to LexA DNA binding domain (Snu114-LexA) showed auto-activation in the Y2H assay when expressed with the pACT2 vector alone (Supplementary Fig. S3). However, 40 mM 3AT inhibited the auto-activation, whereas Snu114-LexA interacted with pACT2-Glc7 under these conditions.

**Figure 2.**
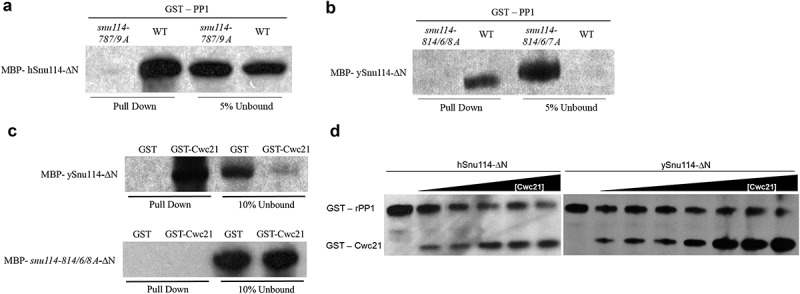
*PP1 and Cwc21 bind Snu114 domain ‘IVa’*. Pull down of purified recombinant MBP tagged (a) hSnu114/*snu114-787/9A-∆N* (b) ySnu114/*snu114-814/6/8A*-∆N with GST tagged rPP1 on glutathione beads. (c) Pull down of MBP tagged ySnu114/*snu114-814/6/8A-∆N* with GST tagged Cwc21 on glutathione beads. The bead-bound proteins were recovered and analyzed by western blot, probing with anti MBP HRP antibodies. GST protein was used as a control for specificity. (d) Pull down of GST tagged rPP1 and/or Cwc21 on Amylose beads. Equal amounts (15 pmol) of rPP1 and hSnu114 or ySnu114 were incubated for 2 h at 4ºC. Increasing amounts (15–170 pmol) of GST-Cwc21 were added and further incubated for 2 hours. Proteins were analyzed by western blot, probing with anti-GST HRP antibodies to identify GST tagged PP1 and Cwc21.

The putative PP1 binding motif in Snu114 is highly conserved from yeast to human (Fig. 1A), and Glc7 has 81% amino acid sequence identity with the rabbit PP1 (rPP1) catalytic subunit [34]. An evolutionary conserved interaction seemed possible. Therefore, to investigate whether Snu114 and PP1 interact directly, we first tested interaction of human Snu114 and rabbit PP1. The N-terminus of Snu114 is strongly acidic, making the recombinant protein relatively insoluble. Therefore, an N-terminally deleted version of hSnu114, (130–972 amino acids) was used. To test the specificity of the interaction, point mutations were made in the ‘RxVxF’ motif of hSnu114 to change the conserved Valine (amino acid 787 (aa787)) and Phenylalanine (aa789) to Alanine (*snu114-787/9-A*). These conserved residues are required in other PP1 interacting proteins for stable PP1 interaction [37,38]. The recombinant hSnu114 (130–972 aa; MBP-hSnu114-ΔN), the mutant derivative (*snu114-787/9A*) and rabbit rPP1 (Full length; GST-PP1) were produced in bacterial cells, purified and tested in a glutathione pull-down assay, where hSnu114-ΔN was pulled down with purified GST-PP1 but not with GST alone (Fig. 2A, data not shown). In contrast, the mutant snu114-787/9A failed to be pulled down by purified GST-PP1 (Fig. 2A), indicating that rPP1 interaction with hSnu114 requires this conserved motif. We also purified recombinant yeast ySnu114 (134-1008aa; MBP-ySnu114-ΔN) as well as a mutant version that has three Alanine substitutions in YGVQYK (*snu114-814/6/8-A*) and performed the pull-down assay with recombinant rPP1. Indeed, rPP1 co-precipitated with wild-type (WT) ySnu114 and not with ySnu114-814/6/8-A (Fig. 2B), indicating that the ‘YGVQYK’ sequence is essential for interaction of ySnu114 with rPP1, and demonstrating the evolutionary conservation of this interaction from yeast to human.

### Cwc21 requires the ‘YGVQYK’ motif to interact with Snu114

We previously demonstrated that Cwc21 interacts directly with a C-terminal fragment of Snu114 (692-951aa [10], within which the ‘YGVQYK’ motif occurs. The PP1 binding motif (the Snu114-Y814-K819) and 777-DTLP-780 region are close to Cwc21-I64-Q111 region in structure immediately after branching (5LJ3 [8], Supplementary Fig. S4). Furthermore, *cwc21-R71D* restricts Cwc21 binding to Snu114 and causes a synthetic growth defect in the absence of Isy1 [10].We therefore tested the effect of the PP1-blocking mutations in the ‘YGVQYK’ motif on the interaction of GST-Cwc21 with MBP-ySnu114, observing that GST-Cwc21 pulled down WT MBP-ySnu114-ΔN but not the MBP-ySnu114-814/6/8-A protein that is mutated in the ‘YGVQYK’ motif (Fig. 2C). We cannot rule out the possibility that the mutant proteins could affect the interactions with PP1 and Cwc21 by a non-specific alteration of the protein structure in vitro, however, their ability to support vegetative growth suggests that they do not cause major structural defects in vivo.

### Cwc21 competes with PP1 for binding to Snu114

To test whether PP1 and Cwc21 compete for binding to Snu114, we performed a pull down of yeast and human MBP-Snu114-ΔN on amylose beads and tested for co-pull down of rPP1 in the presence of increasing amounts of Cwc21. Purified yeast or human MBP-Snu114-ΔN was first incubated for 2 hours at 4°C with a fixed amount of purified GST-PP1, then increasing amounts of purified recombinant GST-Cwc21 were added and the incubation continued for further 2 hours. The bead-bound proteins were analyzed by western blotting with anti-GST antibodies, showing that the presence of increasing amounts GST-Cwc21 resulted in more GST-Cwc21 and less GST-PP1 being pulled down in each case (Fig. 2D), suggesting that Cwc21 and PP1 compete to bind the same region in either yeast or human Snu114.

### Snu114 has higher affinity for Cwc21 when bound to GTP and for rPP1 when bound to GDP

**Figure 3.**
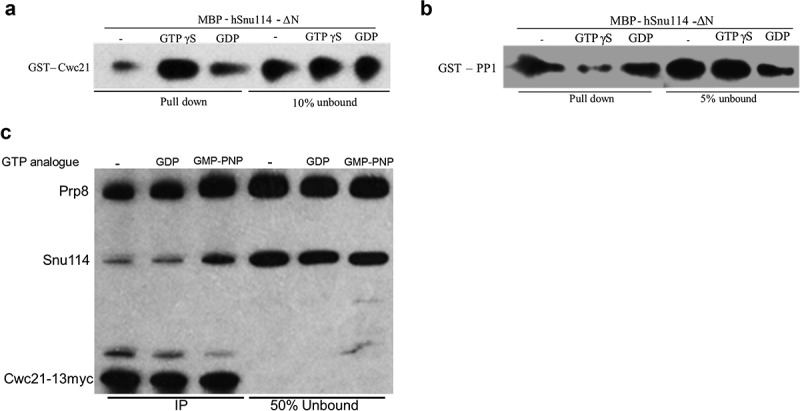
*Snu114 is a GTPase switch*. (a and b) hSnu114 (15 pmol, 45nM) was incubated with 33μM GTPγS/GDP. 15 pmol of Cwc21 or PP1 was added and further incubated before pulling down on Amylose beads. Pulled down proteins were analyzed by western blot probing with anti GST HRP antibodies to identify GST tagged PP1 and Cwc21. Respective pull down protein bands are indicated in the figure. (c) Co-immunoprecipitation of Cwc21-13myc incubated with various GTP analogues. 13myc tagged Cwc21 was immunoprecipitated using anti-myc antibody (9E11) from yeast whole cell extract treated with various GTP analogues. Co-immunoprecipitated proteins were analyzed by western blot probing with anti-Prp8 and anti-Snu114 antibodies.

Snu114 has a GTP binding domain and was proposed to be in different conformations when bound to GTP versus GDP [3]. Considering that Cwc21 and PP1 require similar regions in Snu114 for interaction, we wondered whether the nucleotide bound state of Snu114 affects Snu114 interaction with Cwc21 or rPP1. To test this, we incubated recombinant MBP-hSnu114-ΔN with an excess of GDP or GTPγS (non-hydrolysable analogue of GTP) before incubating with equal amounts of Cwc21 or rPP1. In the pull down assay, Cwc21 associated more with MBP-hSnu114-ΔN in the presence of GTPγS compared with GDP or with no added nucleotide (Fig. 3A), whereas rPP1 associated more with MBP-hSnu114-ΔN in the presence of GDP than with GTPγS (Fig. 3B). This suggests that Snu114 has higher affinity for Cwc21 when bound to GTP and for PP1 when in the GDP bound state. As Cwc21 also interacts directly with Prp8 [10], we performed a similar test using a yeast cell extract in which Cwc21 was 13myc-tagged, and to which GDP or the non-hydrolysable GTP analogue GMPPNP was added. Cwc21-13myc was observed to co-immunoprecipitate more Snu114 as well as more Prp8 in extract to which GMPPNP was added compared with extract to which GDP or no nucleotide was added (Fig. 3C).

### Mutations in Snu114 domain ‘IVa’ cause a defect in entry into meiosis

To determine whether these *snu114* alleles cause defects in splicing we monitored splicing of *ACT1, TUB1 and TUB3* transcripts in strains with *snu114-777DTLP-AAAA* or *snu114-814/6/8-A* mutation in combination with *isy1∆*. As there was no significant accumulation of *ACT1, TUB1* or *TUB3* pre-mRNA or lariat species nor reduction in mRNA levels in these mutants at 37°C (data not shown), the observed growth defects of these *snu114* mutants in the absence of *ISY1* are apparently not due to a general splicing defect but could result from a defect in splicing specific transcripts.

**Figure 4.**
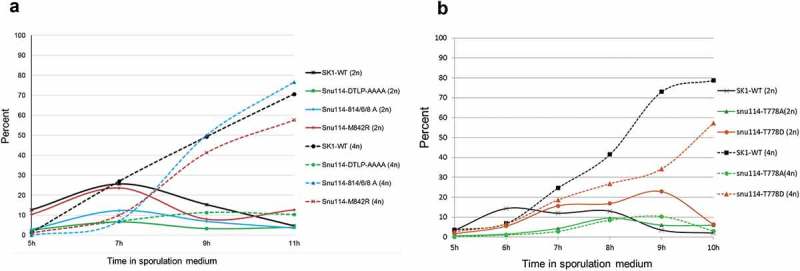
*Analysis of the effects of snu114 mutations on meiosis*. (a) Graph showing the effect of Snu114’s C-terminal mutations on meiosis (n = 300 yeast cells for each time point). (b) Graph showing the effect on meiosis of *snu114* substitution mutations at residue 778 (average data from 2 experiments are shown, n = 600 yeast cells for each time point). Diploid SK1 yeast containing the indicated *snu114* alleles were induced to undergo synchronous meiosis, and samples were harvested at the indicated times and stained with DAPI to assess meiotic progression. The percentage of bi-nucleate (solid lines) and tetra-nucleate (dotted lines) cells is shown with respect to time in sporulation medium. Individual constructs are indicated on the right side of graphs.

As the expression of certain meiosis specific genes is regulated by splicing [39] we investigated the possibility that *snu114* mutations might affect meiosis. SK1 strains of *S. cerevisiae* are commonly used for studying meiotic progression as they sporulate rapidly and synchronously. Therefore, we constructed a diploid SK1 *snu114Δ/snu114Δ* strain carrying *SNU114* on pRS316 (CEN, *URA3*) plasmid. Plasmids (pRS313; *CEN HIS3)* containing the *SNU114-WT, snu114-777DTLP-AAAA snu114-814/6/8-A* or *snu114-40* alleles were introduced into this diploid strain, the *SNU114-URA3* plasmid was counter-selected by growth on 5-fluoro-orotic acid (5-FOA), and the resulting strains were used to monitor the effect of these mutations on meiotic progress. In the presence of WT *SNU114* meiosis proceeded normally, with 70% of cells being tetra-nucleate after eleven hours in sporulation medium (Fig. 4A). Although, *snu114-814/6/8-A* mutant produced a similar level of spore tetrads in eleven hours, sporulation seemed delayed, and was slightly less efficient with *snu114-40* also. More strikingly, the *snu114-777DTLP-AAAA* cells displayed a substantial defect in meiosis, with less than 20% of cells forming tetrads in eleven hours.

‘Phosphomotif finder’ identified a Threonine residue in the evolutionarily conserved sequence DTLP (amino acids 777 to 780 in ySnu114; Fig. 1A) as a likely candidate for phosphorylation. We reasoned that if the Threonine residue in the DTLP motif is indeed subject to phosphorylation, its replacement by Alanine, which inhibits phosphorylation, might account for the meiotic defect of the *snu114-777DTLP-AAAA* mutant. We therefore monitored meiotic progress in mutants *snu114-T778A* and *snu114-T778D* that have single residue substitutions at this position, that inhibit and mimic phosphorylation, respectively. Significantly, the *snu114-T778A* strain failed to produce more than 10% of either bi-nucleate or tetra-nucleate cells, even after 10 hours in sporulation medium, whereas the *snu114-T778D* strain produced almost 60% tetra-nucleate cells in the same period (Fig. 4B). With the *snu114-T778D* strain, sporulation was slow compared to WT, and bi-nucleate cells accumulated between 7 and 9 hours, indicating a defect before the second meiotic division (Fig. 4B).

### A mutation in the C-terminus of Snu114 affects the splicing of meiotic transcripts

**Figure 5.**
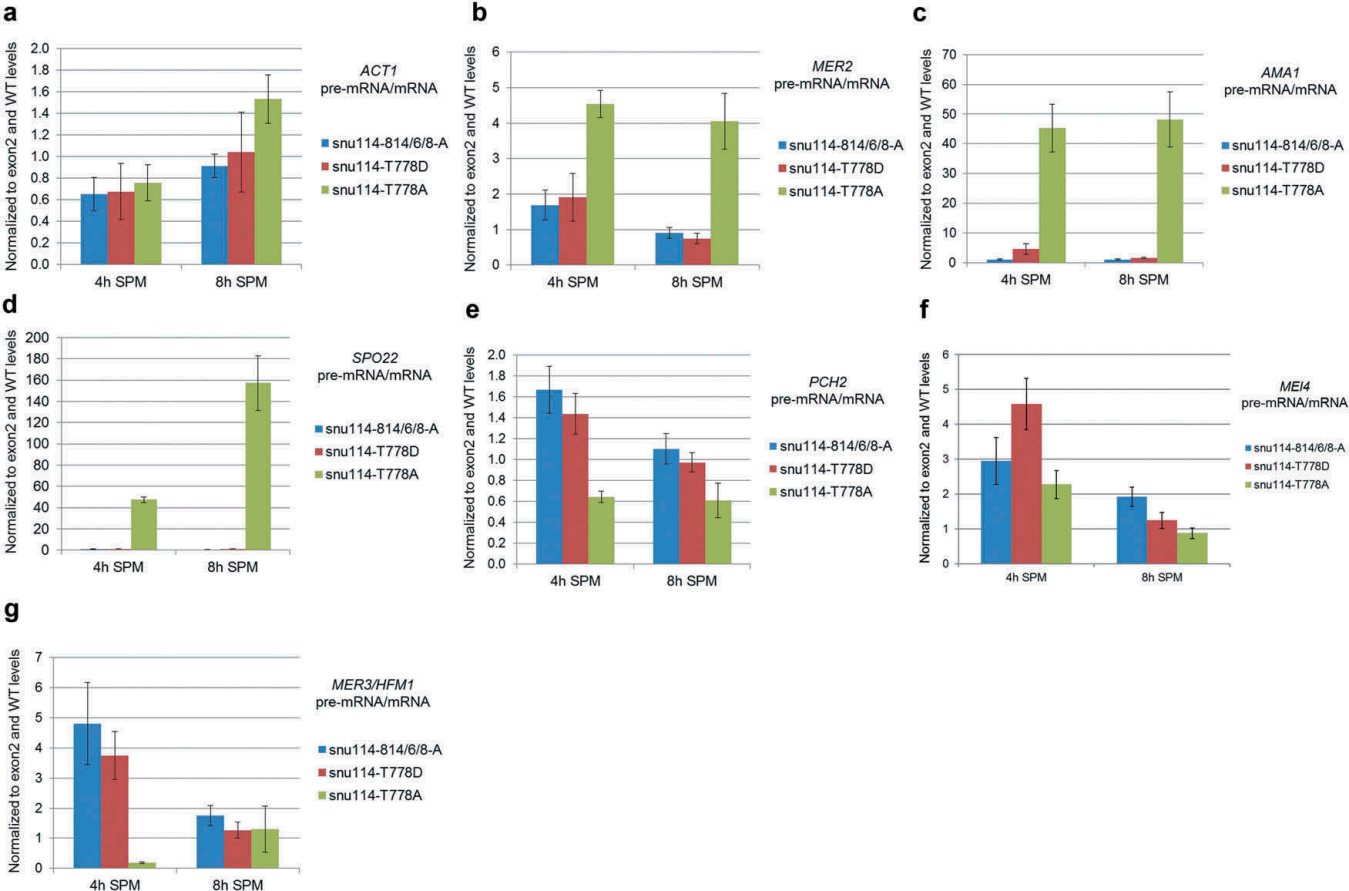
*Snu114-T778A causes inefficient splicing of MER2, AMA1 and SPO22 transcripts*. RT-qPCR analysis of transcripts from (a) *ACT1*, (b) *MER2*, (c) *AMA1*, (d) *SPO22*, (e) *PCH2*, (f) *MEI4*, (G) *MER3*. RNA was extracted from spores after 4 hours and 8 hours in SPM (Sporulation medium, 1% (w/v) potassium acetate, 0.002% (w/v) raffinose). Data information: The data were first normalized to exon 2 (total transcript level) and then to WT *SNU114*. The pre-mRNA/mRNA is shown. Data are presented as mean ± SEM (Standard Error of the Mean) of two biological and six technical replicates.

To determine whether these *snu114* alleles affect splicing during meiosis we monitored the expression of thirteen intron-containing transcripts that were previously shown to be regulated by splicing during meiosis [39]. RNA was extracted from *snu114-T778A, snu114-T778D* and *snu114-814/6/8-A* as well as *SNU114 *WT cells after 4 or 8 hours in sporulation medium, and analyzed by reverse transcriptase-quantitative PCR (RT-qPCR). In a preliminary analysis, the levels of all 13 RNAs (shown previously to be regulated during meiosis [39]) were compared in the various strains by electrophoresis in a Shimadzu bioanalyzer (e.g. Supplementary Fig. S5). Six genes (*AMA1, SPO22, MER2, MER3, MEI4, PCH2*) that showed reduced mRNA level in presence of the *snu114-T778A* allele were selected for more detailed analysis, along with *ACT1* as a control gene that is not subject to meiotic regulation. The pre-mRNA and mRNA levels in the mutants were normalized to exon 2 (total RNA) and WT levels such that a ratio of pre-mRNA/mRNA above 1 indicates a splicing defect. Splicing of *ACT1* transcripts was relatively unaffected by the *snu114* mutations (Fig. 5A; pre-mRNA:mRNA ratios ranging from 0.7 to 1.6 in sporulation medium). *MER2* showed a pre-mRNA:mRNA ratio in the *snu114-T778A* strain approximately 4-fold above WT (Fig. 5B), with the level of pre-mRNA being 3- to 4-fold higher after 8 hours in sporulation medium (Supplementary Fig. S6B), whereas the levels of both pre-mRNA and mRNA in *snu114-T778D* and *snu114-814/6/8-A* were similar to WT. *AMA1* and, especially, *SPO22* transcripts showed a strong splicing defect during meiosis in the *snu114-T778A* strain (Fig. 5(C, D)), with pre-mRNAs accumulating and mRNAs depleted (Supplementary Fig. S6(C, D)). In contrast, *snu114-T778A* did not show accumulation of *PCH2, MEI4* or *MER3* pre-mRNAs nor decrease in their respective mRNAs (Fig. 5(E, G); Supplementary Fig. S6(E, G)). Therefore, the *snu114-T778A* allele specifically affects the splicing of *MER2, AMA1 and SPO22* transcripts. In addition, *snu114-814/6/8-A* and *snu114-T778D* showed accumulation of *MEI4* and *MER3* pre-mRNAs after 4 hours in sporulation medium which was reduced after 8 hours (Fig. 5(F, G)).

It was reported that deletion of either *MER3* or *SPO22* results in delayed transcription of *NDT80*, the transcriptional activator of middle meiotic genes [40]. We therefore measured *NDT80* transcript levels in the *snu114* mutants, finding the level much reduced in the *snu114-T778A* strain by 4h in sporulation medium (Supplementary Fig. S7).

## Discussion

### Snu114 domain ‘IVa’: a substrate/regulatory subunit for PP1

Previous reports suggest the motifs (RK)-x(0,1)-V-x-F and F-x-x-(RK)-x-(RK) as consensus sequences for the recognition and binding of substrates and/or regulators of PP1 [37,41], although the possibility of variations exists. We present here novel results showing PP1 binding to the ‘YGVQYK’ sequence. Our results support the evidence reported by Shi et al [33] for U5-116K (hSnu114) being a substrate for PP1. They showed that hSnu114 gets phosphorylated in the spliceosome and proposed that the dephosphorylation of U2 and U5 specific components by PP1/PP2A leads to structural rearrangements in the spliceosome between the first and second steps in splicing. As these proteins and the mechanism of splicing are highly conserved, it is likely that the proposed phosphorylation event is conserved from humans to yeast. Significantly, phosphorylation of the structurally related eIF-2α (eukaryotic translation initiation factor, also a GTPase) during translation initiation impairs the recycling of eIF-2 [42]. Here, dephosphorylated eIF-2 is active, suggesting regulation through phosphorylation and dephosphorylation during translation initiation. Similarly, the activity of spliceosomal Snu114 may be regulated through reversible phosphorylation. Alternatively, Snu114 may act as a regulatory/targeting subunit of PP1 just as *NIPP1* (Nuclear inhibitor of PP1) acts as a targeting subunit to PP1 for dephosphorylation of the human U2 specific SF3b155 protein [43].

### Cwc21 and PP1: regulator for Snu114 activity

The active site of PP1 and the hydrophobic groove through which PP1 interacts with its putative binding motif, such as the one in Snu114, are 20Å apart [44]. Recent cryo-EM structures of the tri-snRNP (5GAM [6]) and of the spliceosome immediately after branching (5LJ3 [8]) suggest that Snu114-814/6/8 (PP1 binding motif) and Snu114-T778 are less than 20Å apart (Supplementary Fig. S2). Indeed Snu114-K819 forms hydrogen bond with Snu114-D777 (Supplementary Fig. S2). Therefore, it is conceivable that interaction of PP1 and/or Cwc21 with Snu114 controls entry into meiosis. A possible delay in sporulation with the *snu114-814/6/8-A* mutant strain (Fig. 4A) suggests that Snu114 when bound to PP1 affects an event that is important for progress into meiosis. Multiple cycles of phosphorylation and dephosphorylation may be necessary for progression into meiosis as *snu114-T778D* (which mimics phosphorylation) does not reach WT levels of sporulation (Fig. 4B). With the *snu114-814/6/8-A* mutant strain, the inability of PP1 to bind Snu114 may affect the multiple phosphorylation/dephosphorylation cycles, causing the observed delay in sporulation, although direct evidence of this is lacking.

As the *snu114-T778A* mutation within the DTLP motif causes a sporulation defect, it is a potential downstream target of PP1. Conceivably, when PP1 is bound to Snu114 at position 814–818 it might prevent the downstream kinase (possibly Casein Kinase 2, see below) phosphorylating threonine 778. The displacement of PP1 by Cwc21 would then allow ‘T778ʹ to be phosphorylated, permitting meiosis to proceed. In such a scenario T778 phosphorylation occurs only after PP1 is displaced or prior to its association with Snu114. In the cryo-EM structure of the budding yeast post catalytic spliceosome [14] Cwc21 spans part of pre-mRNA exon1 and around the 777-DTLP-780 region in Snu114 domain IV. The genetic interactions in Bai et al [14], suggest functional importance for this interaction. Cwc21 and another splicing factor Cwc22 are required to stabilize binding of exon 1 to U5 snRNA loop 1 [14]. This suggests a possible role for Cwc21 and/or Isy1 in regulating Snu114’s function in meiotic splicing.

### Cwc21 and PP1: effectors for Snu114

Although it was proposed that GTP hydrolysis by Snu114 is important for splicing [3,5,45], recent results have challenged the evidence that Snu114 has GTPase activity or that it is required for growth [6]. Our results now raise the question of whether ‘GTP/GDP exchange or GTP hydrolysis by Snu114 may have a role in meiotic splicing. The observed preference for Cwc21 and PP1 for binding Snu114 according to its GTP/GDP bound state, supports the hypothesis [3,5] that Snu114 domain ‘IVa’ has different conformations in its different nucleotide bound states, like the GTPase EF-2 (translation elongation factor). In addition, these findings suggest possible regulatory roles for Cwc21 and PP1, functioning as effector proteins for Snu114 during nucleotide exchange and/or GTP hydrolysis.

During translation initiation, eukaryotic initiation factor 5 (eIF5) acts like a classical GAP [46] to facilitate GTP hydrolysis by eIF2. eIF5 possesses a conserved arginine (Arg15) contributing to catalysis similar to the arginine finger of classical GAP’s, which are flanked by hydrophobic residues. In addition, Arg48 of eIF5 stabilizes the transition stage of GTP hydrolysis in the eIF2.eIF5 complex [46]. Interestingly, Cwc21 also contains highly conserved arginine residues Arg71 and Arg103. It is likely that Arg71 in Cwc21 contributes to the stable interaction of Cwc21 with Snu114. Mutation of Arg71 to Aspartate restricts Cwc21 binding with Snu114 and causes a synthetic growth defect at 37ºC in the absence of Isy1 [10]. Furthermore, Cwc21 (I64-Q111), which contains R71, is very close to Snu114 domain IVa in the cryo-EM structure of the spliceosome immediately after branching (5LJ3 [8] Supplementary Fig. S3).

The nucleotide binding sites of ‘G’ proteins are modified upon binding of GEF proteins [47], such that the affinity for GDP is reduced and it is replaced by GTP. Interaction of PP1 with the C-terminus of Snu114 may regulate exchange of GDP to GTP. Competition between PP1 and Cwc21 for interacting with Snu114 would then determine the active state of Snu114 in the spliceosome. Phosphorylation of eIF-2α impairs the recycling of eIF-2 by affecting the GDP/GTP exchange [42]. Likewise, phosphorylation of Snu114 may affect its nucleotide exchange, with PP1 being required to dephosphorylate Snu114 for efficient nucleotide exchange.

### Snu114: novel role for non canonical pre-mRNA transcript stabilization

Splice site sequences are highly conserved among intron-containing genes of budding yeast that are expressed during vegetative growth, ensuring their efficient recognition by the splicing machinery. However, several meiosis-specific genes contain introns with non-canonical sequences and the transcripts are spliced only during meiosis, despite some being present also in vegetatively growing cells. *MER1* encodes a U1 snRNP-associated protein that is produced early during meiosis [27,28], and is required for the splicing of four meiosis-specific transcripts, *MER2 (REC107), MER3 (HFM1), SPO22* and *AMA1 (SPO70)* [29]. Nam8, another U1 snRNP protein, is required for Mer1 function during meiosis [28,29], but it is expressed during both vegetative growth and meiosis, and is also required for splicing *PCH2* transcripts [48]. Similarly, RES (Retention and Splicing) complex protein Snu17/Ist3 is required for *MER1* function whereas Bud13 from the same complex is required for *MER1* activated *AMA1* and *MER2* splicing but not for *MER3* splicing [49].

In striking contrast to previously characterized *snu114* mutations that seem to inhibit splicing generally in mitotically dividing cells, our alanine-scanning mutagenesis of domains IVa and IVb uncovered a novel, allele-specific defect in meiotic splicing. The meiotic defect caused by the *snu114-T778A* allele is, at least in part, a consequence of inefficient splicing of three Mer1- and Nam8-dependent transcripts, *MER2, SPO22* and *AMA1*. Mer2 is a meiosis-specific protein involved in the initiation of recombination through formation of double strand breaks [50]. Spo22 on the other hand is essential for chromosome synapsis [51]. Ama1 activates the meiotic anaphase promoting complex and is also required for spore wall formation [52,53]. Deletion of either *MER3* or *SPO22* [40] results in a delay at the pachytene checkpoint and delayed expression of *NDT80*, the transcriptional activator of middle meiotic genes. Indeed, we observed that poor splicing of *SPO22* (Fig. 4) correlates with reduced *NDT80* expression in the *snu114-T778A* mutant (Supplementary Fig. 7), compatible with the requirement for optimum levels of Spo22 for *NDT80* expression. Furthermore, we observed inefficient splicing of *MER3* in *snu114-814/6/8-A* and *snu114-T778D* alleles after 4 hours in sporulation medium (Fig. 5(F, G)) which could possibly contribute to delayed sporulation in these alleles (Fig. 4).

In our study, the *snu114-T778A* mutation affects only *SPO22, AMA1* and *MER2*. The mutation affects a predicted target for phosphorylation (see below) in Snu114 and, conceivably, residue T778 may have to be phosphorylated for optimal splicing of these transcripts. As *SPO22, AMA1* and *MER2* have non-canonical 5’SS sequences and the exonic bases upstream of the 5’SS are not optimal for base-pairing with U5 loop 1, assembly in spliceosomes and alignment of the exons may be error-prone. Therefore, Snu114 might promote their splicing by supporting Mer1 and Nam8 in stabilizing their 5’SS:U1 snRNP interactions prior to Brr2 function, or Snu114 may act with Prp8 to stabilize the 5ʹexon:U5 snRNP interactions in the catalytic center, with these roles being compromized in the *snu114-T778A* mutant. Alternatively, Snu114 may affect proofreading of these suboptimal interactions by one of the spliceosomal ATPases that effect quality control [54,55]. However, for optimal splice site sequences, T778 may have to be dephosphorylated such that the base pairing (5ʹexon:U5 snRNP) is not hyperstabilized. Hence, the phosphorylation status of Snu114 C-terminus (T778) could determine the optimum stabilization of U5 loop 1 and exonic bases upstream of 5’SS. Interestingly, in the human lung adenocarcinoma cell line PC9 derivative PC9/gef clones Snu114-T749 (T778 in yeast Snu114) is phosphorylated upon Casein Kinase 2 (CK2) treatment [56]. In addition, Snu114-T749 (T778 in yeast Snu114) is also phosphorylated in an immortalized human T lymphocyte cell line (Jurkat cells) (https://www.phosphosite.org/curatedInfoAction.caction?record= 22,693,600 [57]). This is compatible with the functionally important role for yeast Snu114-T778 phosphorylation.

How would phosphorylation at this position influence Snu114 function? To investigate this we looked into the molecular components of tri-snRNP (PDB ID: 5GAM [6], MMDB ID: 136,295) and the spliceosome structure immediately after branching (PDB ID: 5LJ3 [8], MMDB ID: 141,626). We modified these structures in Swiss PdvViewer to investigate how phosphomimic and dephosphomimic mutations could theoretically affect the stability of the structure. In the foot region of the tri-snRNP structure (5GAM [6]) residue T778 forms hydrogen bonds with K792 and V765 (Supplementary Fig. S8(A)). However, the alanine mutant A778 would appear to prevent hydrogen bonding with either K792 or V765 (Supplementary Fig. S8(B)). In contrast, substitution with aspartic acid, D778 is predicted to stabilize the interaction through a hydrogen bond with Serine 789 (Supplementary Fig. S8(C)). Similarly, in the structure immediately after branching (5LJ3 [8]) T778 forms a hydrogen bond with K792, which is absent when mutated to alanine but forms double hydrogen bonds with S789 when mutated to aspartic acid (Supplementary Fig. S8(D, F)). In both cases the phosphomimic mutation is predicted to be more stable compared to the de-phoshphomimic mutation (T778A is less stable than T778D). As other meiosis-specific introns are also characterized by unusual intron sequences, a more in-depth analysis will be required to determine what makes the *SP022, AMA1* and *MER2* introns particularly sensitive to the *snu114-T778A* allele. Nonetheless, this finding is remarkable in identifying a new role for Snu114 in meiotic splicing that, considering the high conservation of this protein, may be important during development in higher eukaryotes more generally.

## Materials and methods

### Strains and plasmids

All strains and plasmids used in this study are listed in supplementary table T2 and supplementary table T3, respectively. Deletion of *SNU114, CWC21* and *ISY1* from the genome was performed using one step PCR [58], with the essential *SNU114* gene carried on plasmid pRS316. Mutant *snu114* alleles were introduced on pRS313 or pRS315 followed by plasmid shuffle in presence of 5-FOA to eliminate the pRS316 plasmid. Colony PCR was performed to confirm the correct replacement of *SNU114*. These SK1 cells were sporulated and dissected using a Micromanipulator (Singer Instruments MSM System). The mating type was identified and haploid SK1 ‘a’ and ‘α’ cells with *SNU114* deletion were mated to produce a homozygous *snu114* knockout SK1 strain. pRS313-*snu114-777DTLP-AAAA* and pRS313-*snu114-814/6/8-As* were then introduced into this diploid SK1 by plasmid shuffle.

### Yeast two-hybrid assay

The Y2H system utilizes the interaction between two fusion proteins, one containing the LexA-DNA binding domain (DB; expressed from pBTM116), the other containing the Gal4-activating domain (AD; expressed from pACT2), to control the expression of a LexAop-*HIS3* reporter gene. Yeast strain L40∆G was transformed with the prey-plasmid (DB) and selected on – L (without Leucine) growth medium, followed by a bait-plasmid (AD), selected on – W (without Tryptophan). To test for protein interaction, which results in induction of the reporter gene, the cells were spotted on -L-W-H (without Leucine, Tryptophan and Histidine) plates with various concentrations of 3-amino-1,2,4-triazole (3-AT). 3-AT is a competitive inhibitor of histidine biosynthesis and inhibits any basal transcription of the reporter gene.

### Recombinant protein purification

Gateway system (Inivtrogen) was used as per manufacturer’s instruction for preparing recombinant plasmids for protein expression. A modified version of Stratagene ‘Quick Change protocol’ was used to mutate the plasmid-encoded *SNU114*. Recombinant plasmids were transformed either in *Escherichia coli* Rosetta™ 2 or *E. coli* T7 Express *I^q^*. The cells were induced with 0.4 mM IPTG (Invitrogen). For lysis, a cell disrupter (Constant Systems Limited) was used at 22 KPSI in lysis buffer (50 mM HEPES pH 7.5, 500 mM NaCl, 10 mM MgCl_2_, 10 mM β-Me, 10 mM imidazole, 3 cOmplete™ mini protease inhibitor cocktail tablet). The cell lysate were then affinity purified using Amylose/Glutathione agarose beads in Biorad Column. The proteins were eluted with 10 mM Maltose (Sigma) for MBP tagged proteins and 40 mM Glutathione (Fluka) for GST tagged proteins. The eluted protein was further purified by gel filtration using a size exclusion column (Hi Prep™ 16/60 Sephacryl™ S-200, High resolution) in a HPLC apparatus (ÄKTA systems). The proteins were dialyzed against 20 mM HEPES at pH 7.5, 100 mM NaCl, 100 µM EDTA, 1 mM DTT, 20% glycerol. Protein concentration was determined using Bradford assay. All buffers used during GST-rPP1 purification contained 1 mM MnCl_2_.

### Recombinant protein pull down assay and western blotting

15 pmol of each protein were incubated with 200 µl of ice-cold X1 IP_150_ (6mM HEPES pH 7.9, 150mM NaCl, 5mM MgCl_2_) by rotating end-over-end at 4°C for 1 hour. Simultaneously, pre-swollen glutathione/Amylose beads were washed three times with NTN buffer (50mM Tris at pH 7.5, 15 mM NaCl, 0.1% [v/v] Nonidet P40) followed by X1 IP_150_ and resuspended in 100µl of IP_150_ before incubating with proteins for 1–2 hrs at 4°C with end-over-end rotation. The beads were then washed twice with NTN and twice with NT (NTN without NP-40) buffers before resuspending in protein loading buffer (200 mM Tris pH 6.8, 8% (w/v) SDS, 40% (v/v) Glycerol, 40 mM DTT, 0.4% (w/v) bromophenol blue). The bound proteins were denatured at 70°C for 5–10 min and run on pre-cast 4–12% Bis-Tris gel (Novex, Invitrogen) for 1 hr. The gel was blotted on a nitrocellulose membrane using a western blot apparatus (Invitrogen) for 1–2 hrs. Anti-GST (B-14) HRP (Mouse monoclonal IgG, Santa-Cruz) was used at 1/10,000 and anti-MBP HRP (Rabbit monoclonal IgG, NEB) was used at 1/8000 dilution. The membrane was stained using Chemical Luminescence (Santa Cruz Biotech.) and visualized in Kodak general purpose blue films in an X-ray developer (Fujifilms).

### Splicing analysis by real-time quantitative RT-PCR

The method of Schmitt et al [59] was followed for total yeast RNA extraction. RNA was first treated with DNaseI (Promega) and Roche transcriptor was used to generate cDNA as per manufacturer’s instructions. Roche SyBr Green mix was used for the Real Time qPCR on a Stratagene Mx3005P according to manufacturer’s instructions. Relative abundance with respect to a control was achieved by using the formula: Relative abundance = 2^−(ΔCt)^ (where Ct = the threshold cycle and ΔCt = Ct_test_-Ct_control_).

### Yeast sporulation, tetrad dissection and mating

Cultures were grown in Yeast Peptone Dextrose Adenine (YPDA: 2% (w/v) peptone, 1% (w/v) yeast extract, 2% (w/v) glucose, 100 µg/ml Adenine; to make *snu114∆/snu114∆* diploid shuffle strain) or drop out media (to select for plasmids) overnight to stationary phase. 20 ml YPA (pre-sporulation medium, 2% (w/v) peptone, 1% (w/v) yeast extract, 1% (w/v) potassium acetate) cultures were inoculated in 100 ml flask to OD_600_ 0.10–0.15 and grown with good aeration for 13–14 hours. Cultures with OD_600_ between 1.6–3.0 were harvested and washed with H_2_O, re-suspended in 10 ml SPM (Sporulation medium, 1% (w/v) potassium acetate, 0.002% (w/v) raffinose) to an OD_600_ of 1.8–1.9 and incubated at 30ºC shaking vigorously (250 rpm). After 4 hours in SPM, 100 µl of cells were removed every hour and fixed with 400 µl of 95% ethanol. Cell pellets were then stored at 4°C. Tetrads were stained with DAPI and monitored using microscope (Leica DMRA2). For RNA extraction, volumes were scaled up. For tetrad dissection 75 µl cell culture in sporulation medium with an OD_600_ of 2.0, was pelleted and resuspended in 18 µl of 1 M sorbitol, and Zymolyase was added to 1 mg/ml Following incubation for 10 min at 20°C, 400 µl of ice cold water was added and stored on ice until required. Tetrads were dissected using Micromanipulator (Singer Instruments MSM System).

## Data Availability

Most data generated or analyzed during this study are included in this published article (and its Supplementary Information files). The datasets that are not included are available from the corresponding author on reasonable request.
