# Supplementary material for: Mutagenesis of Snu114 domain IV identifies a developmental role in meiotic splicing
Source: RNA Biol. 2019 Jan 23;16(2):185–95. doi: 10.1080/15476286.2018.1561145 (PMC6380292; doi:10.1080/15476286.2018.1561145)
Supplement: Supplemental Material [file krnb-16-02-1561145-s001.pdf]

Mutagenesis of Snu114 domain IV identifies a developmental role in meiotic splicing.

Amit Gautam[1] and Jean D. Beggs

Wellcome Centre for Cell Biology, University of Edinburgh, King’s Buildings,
Mayfield Road, Edinburgh, EH9 3BF, UK.

[1]Current address: Genome Damage and Stability Centre, University of Sussex, Science Park Road, Falmer, Brighton, BN1 9RQ, UK.

Supplementary Figures S1 to S8 and Table T1 to T3

Supplementary Figure S1

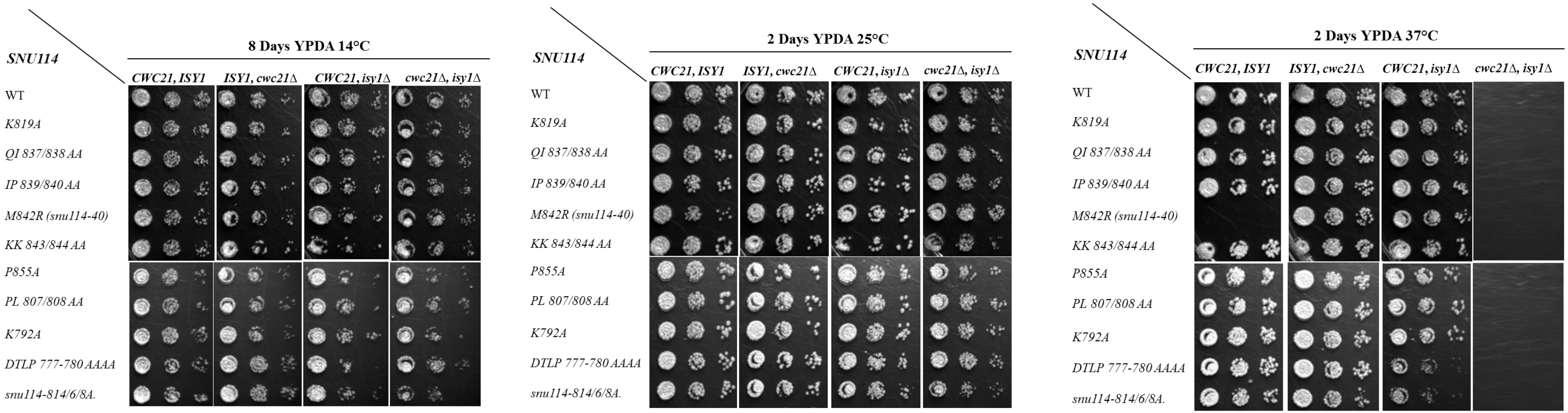

Supplementary figure S1 (related to figure 1). *SNU114* interacts genetically with *CWC21* and *ISY1*. Growth defects observed in plasmid shuffle assay. After shuffling out the wild-type plasmid, yeast cells expressing Snu114 from the mutated plasmids (indicated in the figure) were grown to stationary phase in YPDA medium. Then, cells diluted to OD_{600} of 0.3 were spotted on YPDA plates and grown at 25°C or 37°C for two days or at 14°C for eight days.

Supplementary Table T1

snu114 mutant	Location	Temperature sensitivity	Synthetic Interaction with *CWC21/ISY1*	Reference
777DTLP-As	IVa	No	Yes	This work
K792A	IVa	No	No	This work
PL807-808AA	IVa	No	No	This work
814/6/8 A	IVa	No	Yes	This work
K819A	IVa	No	No	This work
QI837-838AA	IVa	No	No	This work
IP839-840AA	IVa	No	No	This work
M842R (snu114-40)	IVa	37°C	Yes	1, 2
KK843-844AA	IVa	No	No	This work
P855A	IVa	No	No	This work
D943A	IVb	No	No	This work
D946A	IVb	No	No	This work
K947A	IVb	No	No	This work
R964A	IVb	No	No	This work
D965A	IVb	No	No	This work
K969A	IVb	No	No	This work
T970A	IVb	No	No	This work
RRRK971-974AAAA	IVb	No	No	This work
KKRR881-884AAAA	IVb	No	No	This work
D984A	IVb	No	No	This work
K991A	IVb	No	No	This work
snu114-60 (K939Δ)	IVb	14°C and 37°C	Yes	1

Supplementary Table 1 (related to figure 1). *SNU114* interacts genetically with *CWC21* and *ISY1*. Alanine scanning mutations in *SNU114* domains IVa and IVb screened for growth defects in this study.

Supplementary Figure S2

A

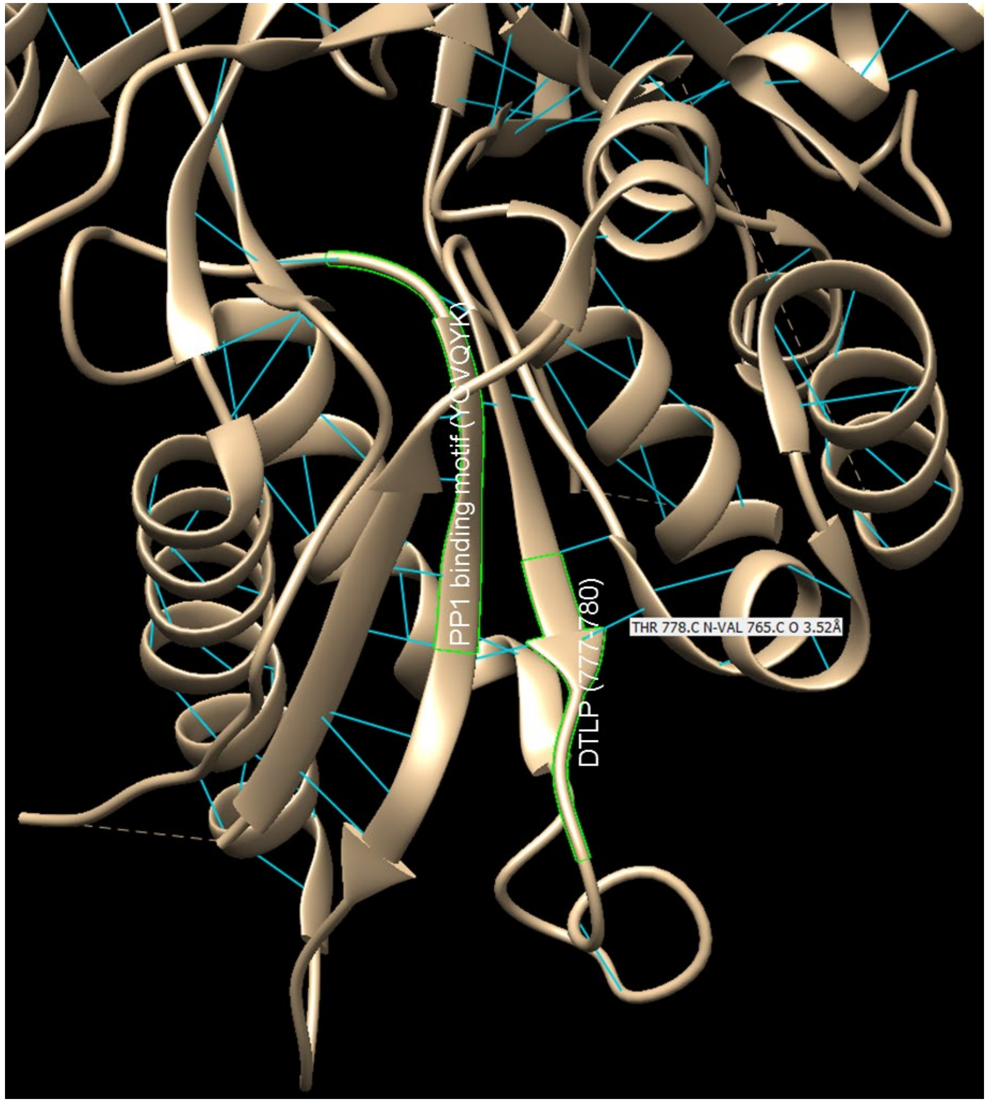

Shows proximity of DTLP and PP1 site in tri-snRNP structure (5GAM[3])

B

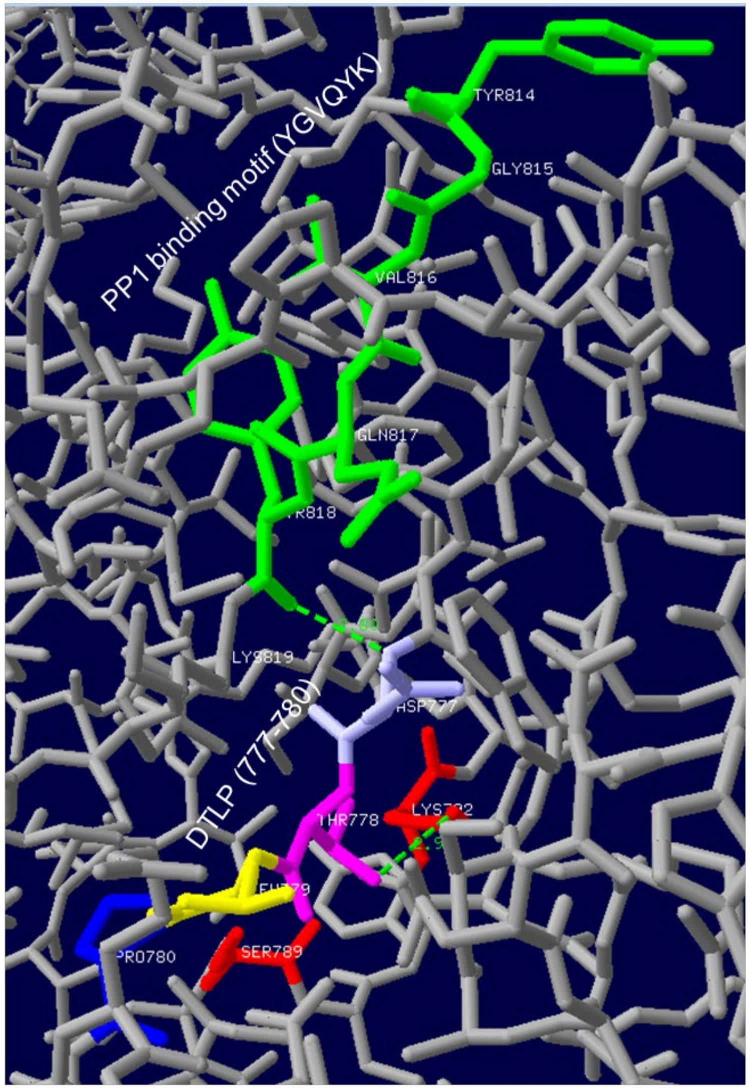

Interaction of Snu114-D777 (Snu114-$_{777}$DTLP$_{780}$) with Snu114-K819 (Snu114-$_{814}$YGVQYK$_{819}$) in structure after branching (5LJ3[4])

Supplementary Figure S2. Proximity of Snu114-DTPL and putative PP1 binding motif in the cryo-EM structures (5GAM[3] and 5LJ3[4]).

Supplementary Figure S3

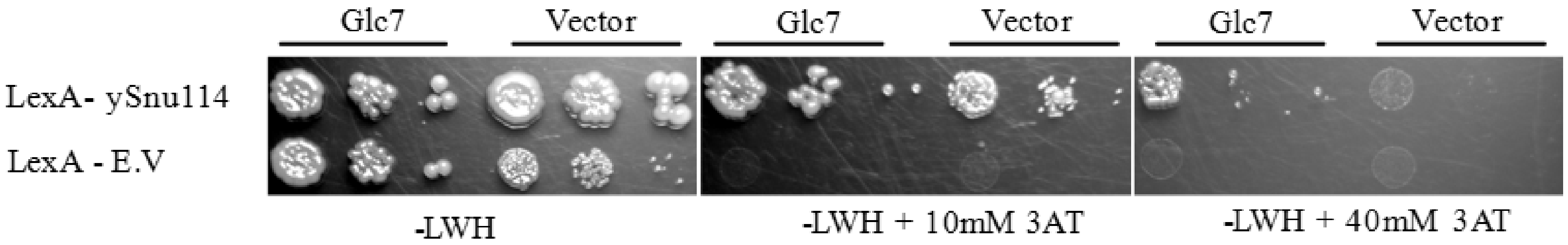

Supplementary figure S3 (related to figure 2). **Yeast two-hybrid interaction of Snu114 and Glc7 (PP1).** Interaction of pACT2-Glc7 (containing activating domain) with full length LexA-Snu114 constructs on –LW (without Leucine and Tryptophan) or –LWH (without Leucine, Tryptophan and Histidine) plates with different concentrations of 3-AT (3-Amino-1,2,4-triazole) as indicated. The cells spotted are diluted to OD = 0.3 and two ten-fold serial dilutions. Plates were incubated at 30ºC for at least 2 days.

Supplementary Figure S4

A

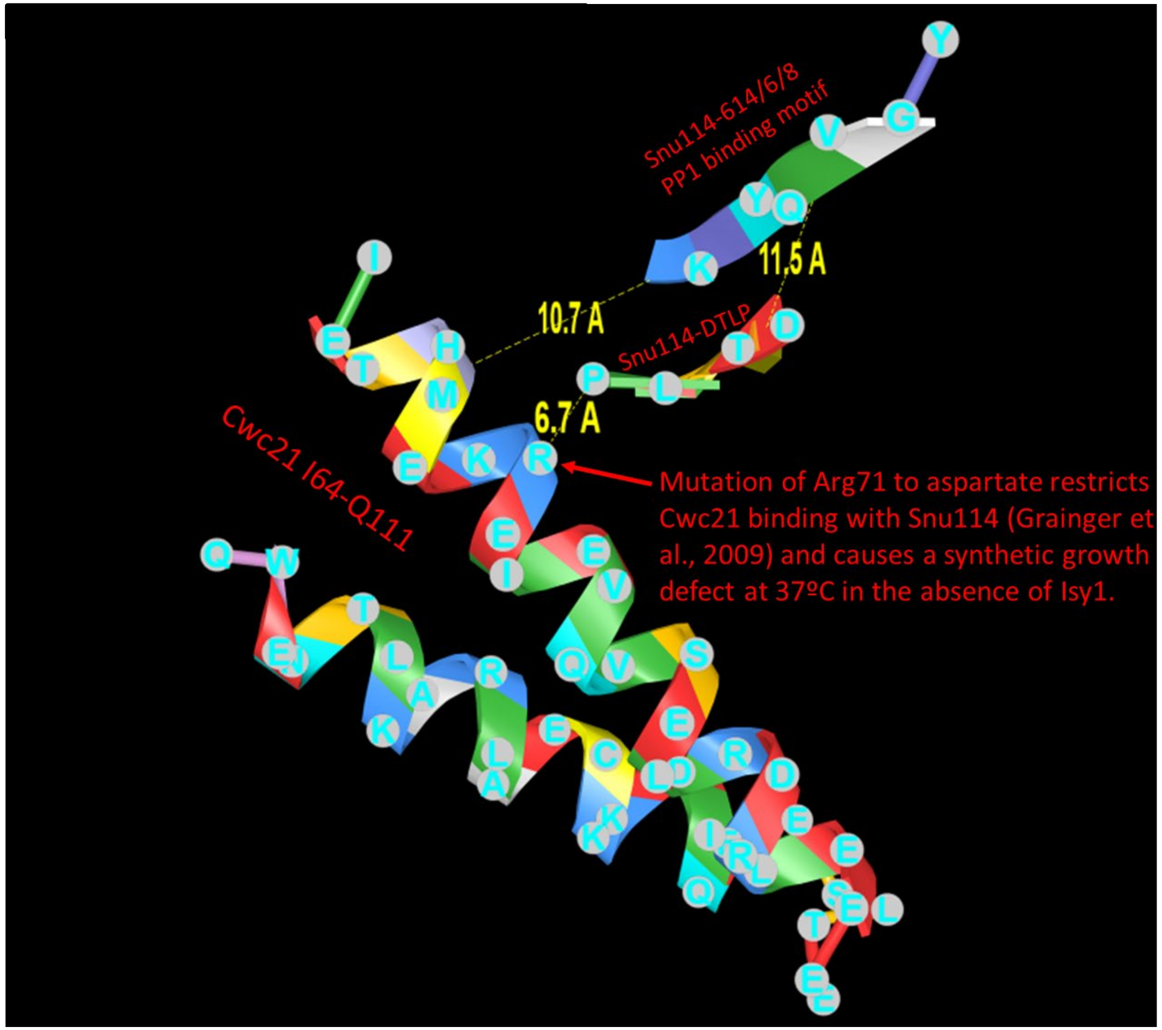

Cwc21 (I64-Q111) proximity to putative PP1 binding motif and DTLP region in Snu114 in the 5LJ3[4] structure (immediately after branching)

B

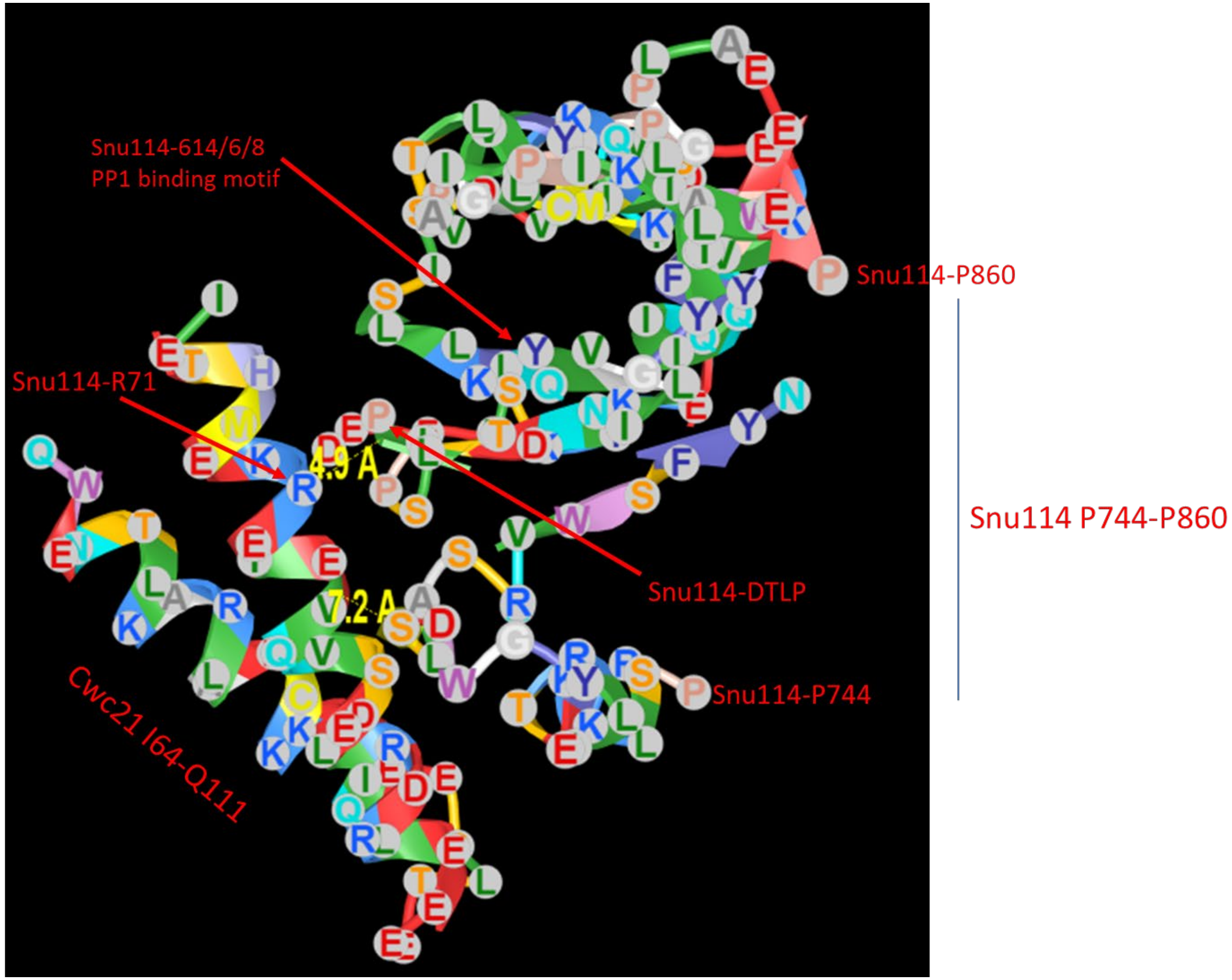

Cwc21 (I64-Q111) proximity to Snu114 domain IVa in the 5LJ3[4] structure (immediately after branching)

Supplementary figure S4. Proximity of Cwc21 (I64-Q111) to both Snu114-DTPL and putative PP1 binding motif in the cryo-EM structures.

Supplementary Figure S5

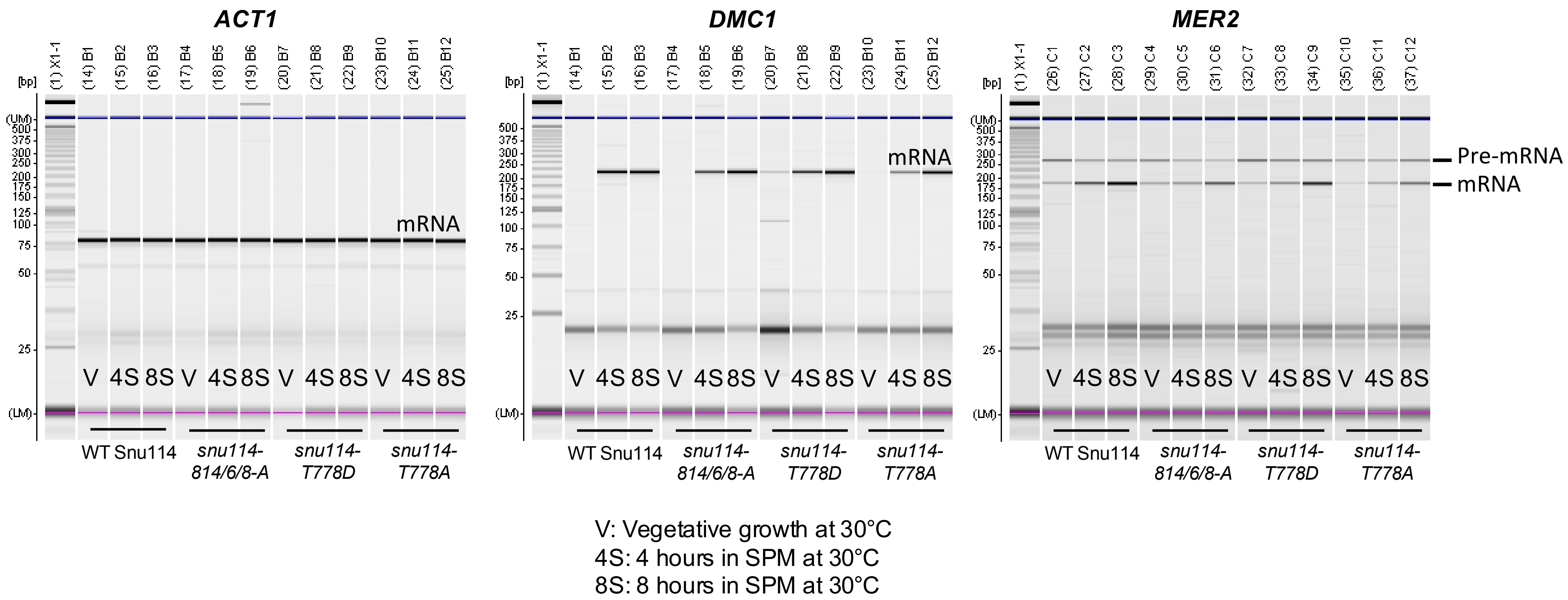

Supplementary figure S5 (related to figure 5). *snu114-T778A* causes inefficient splicing of *MER2* transcript. RT-PCR (25-30 cycles) and gel electrophoresis analysis of *ACT1*, *DMC1* and *MER2* mRNA transcripts in RNA extracted from different *SNU114* mutant strains. The corresponding mRNA bands for *ACT1*, *DMC1* and *MER2* are 82, 216 and 230 nucleotides respectively whereas the upper band observed in *MER2* corresponds to unspliced *MER2* pre-mRNA which is 303 nucleotides long. The strains and growth conditions are indicated on the figure.

Supplementary Figure S6

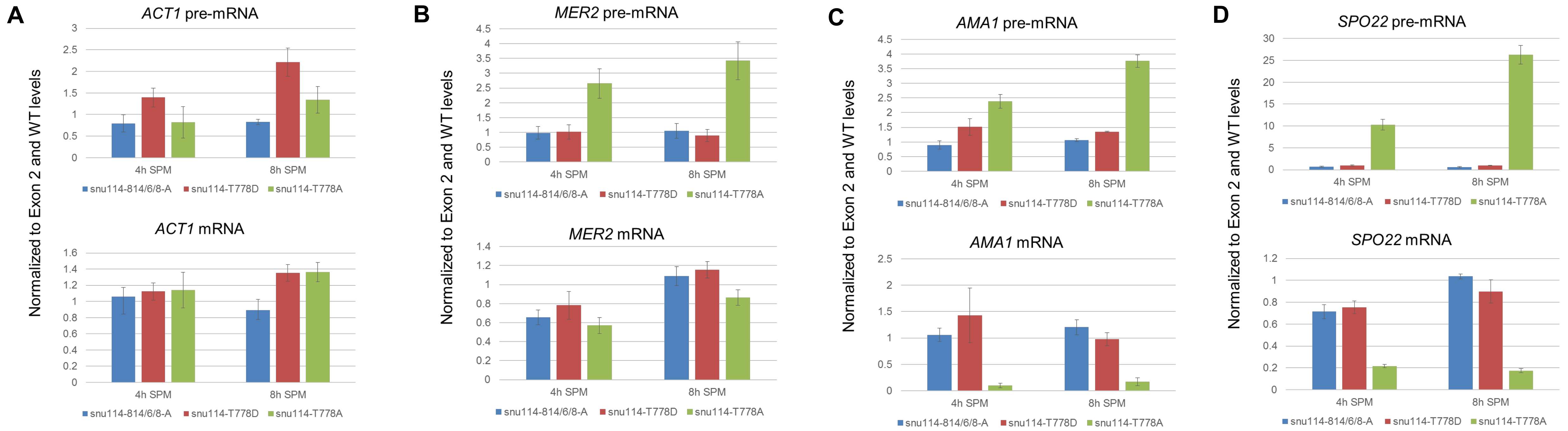

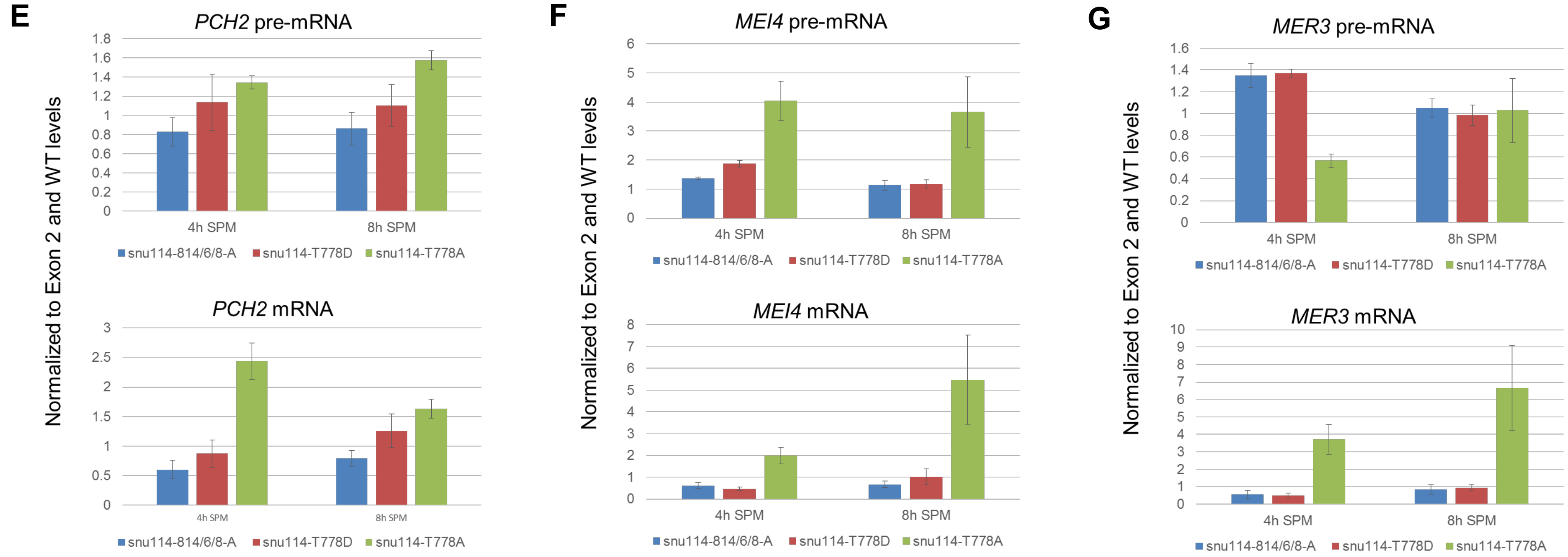

Supplementary figure S6 (related to figure 5). *snu114-T778A* causes inefficient splicing of *MER2, AMA1* and *SPO22* transcripts. RT-qPCR analysis of transcripts from (A) *ACT1,* (B) *MER2,* (C) *AMA1,* (D) *SPO22,* (E) *PCH2*, (F) *MEI4*, (G) *MER3*. RNA was extracted from spores after 4 hours and 8 hours in SPM (Sporulation medium, 1% (w/v) potassium acetate, 0.002% (w/v) raffinose). Data information: The data were first normalized to exon 2 (total transcript level) and then to WT *SNU114*. pre-mRNA and mRNA are shown separately unlike figure 5. Data are presented as mean ± SEM (Standard Error of the Mean) of two biological and six technical replicates. Blue bar (snu114-814/6/8-A), Brown bar (snu114-T778D) and Green bar (snu114-T778A).

Supplementary Figure S7

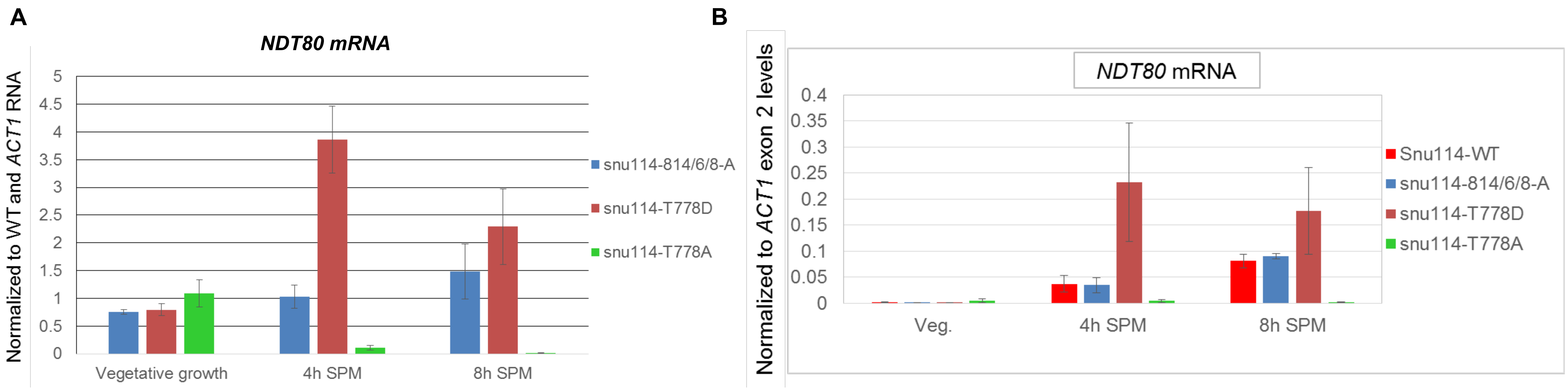

Supplementary figure S7. ***snu114-T778A* causes low *NDT80* expression.** For RT-qPCR analysis of *NDT80*, RNA was extracted from cells during mid-log growth or after 4 hours and 8 hours in SPM. Data information: The data were normalized to WT (A) and *ACT1* exon 2 (B) at the appropriate time in SPM. Data are presented as mean ± SEM of two biological and six technical replicates.

Supplementary Figure S8

A

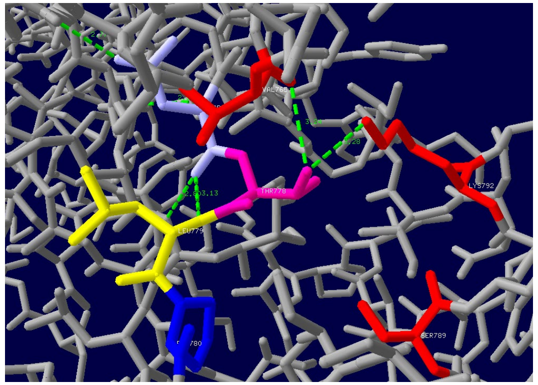

T778 interaction with K792 and V765 in tri-snRNP (5GAM)

B

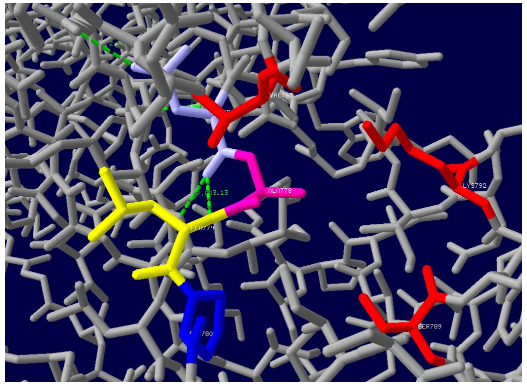

Effect of T778 mutation to Alanine, resulting in loss of interaction with K792 and V765 in tri-snRNP (5GAM)

C

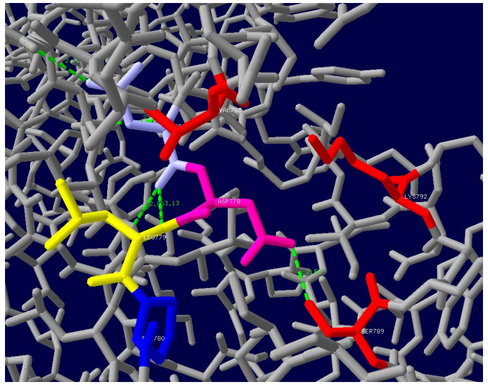

Effect of T778 mutation to Aspartic Acid, interaction now with S789, whereas T778 showed interaction with K792 and V765 in tri-snRNP (5GAM)

D

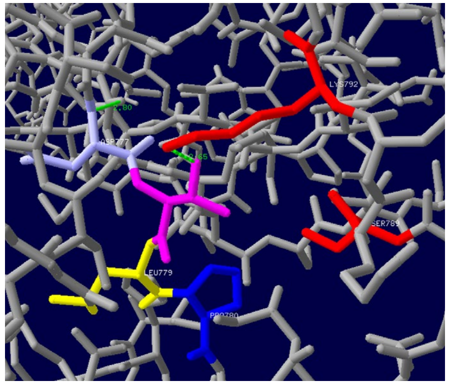

T778 interaction with K792 in structure after branching (5LJ3)

E

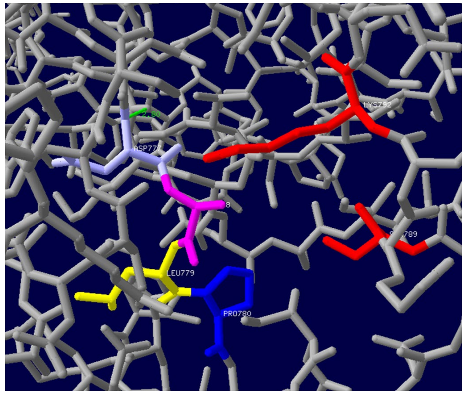

Effect of T778 mutation to Alanine, resulting in loss of interaction with K792 in structure after branching (5LJ3)

F

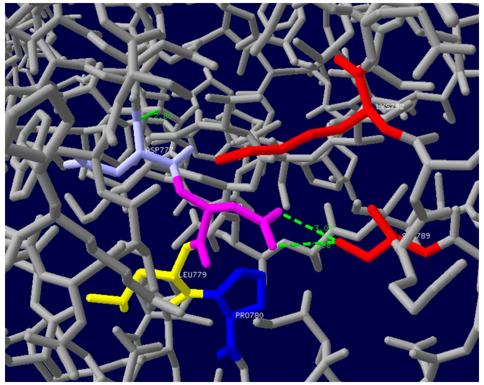

Effect of T778 mutation to Aspartic Acid, interaction now with S789, whereas T778 showed interaction with K792 in structure after branching (5LJ3)

Supplementary figure S8. Stability of Snu114 residue T778 is compromised upon de-phosphomimic mutation. Modified 5GAM[3] and 5LJ3[4] structures to show the effect of phosphomimic (Aspartic acid) and de-phosphomimic (Alanine) mutation on Snu114 residue T778. The coordinates were imported in Swiss-Pdb viewer and hydrogen bonds in the relevant residues highlighted. To access hydrogen bonds in mutant residues, the amino acid was modified in Swiss-Pdb viewer.

Supplementary Table T2

Strain	Genotype	Source/reference
L40 ΔG	*MAT**a** his3Δ200 trp1-901 leu2-3, 112 ade2 LYS2::(4lexAop-HIS3) URA3::(8lexAop-lacZ) gal4Δ::KAN*	5
SK1 (2n)	*Mata/α, ho:LYS2/ho:LYS2, lys2/lys2, ura3/ura3, leu2::hisG/leu2::hisG, his3::hisG/his3::hisG, trp1::hisG/trp1::hisG,*	A gift from Adele Marston
SK1 (2n) Snu114 shuffle strain	*Mata/α, ho:LYS2/ho:LYS2, lys2/lys2, ura3/ura3, leu2::hisG/leu2::hisG, his3::hisG/his3::hisG, trp1::hisG/trp1::hisG, snu114Δ::Hph/snu114Δ::Hph,* pRS316 *SNU114*	This work
yTB23	*MATa, lys2Δ, his3Δ, leu2Δ, ura3Δ, met15, snu114::KanMX, pTB1*	1
yRG23	*MATa, lys2Δ, his3Δ, leu2Δ, ura3Δ, met15, snu114::KanMX, pTB1, cwc21::HPH-NT1*	2
yTB23b	*MATa, lys2Δ, his3Δ, leu2Δ, ura3Δ, met15, snu114::KanMX, isy1::natNT2 pTB1*	This work
yRG23b	*MATa, lys2Δ, his3Δ, leu2Δ, ura3Δ, met15, snu114::KanMX, pTB1, cwc21::HPH-NT1, isy1::natNT2*	This work

Supplementary Table T2. Source and genotype of strains used in this study.

Supplementary Table T3

Name	Description	Source
pFA6a-hphNT1	Hygromycin knockout vector	PCR toolbox, Euroscarf
pFA6a-natNT2	Noourseothricin knockout vector	PCR toolbox, Euroscarf
pRS313	*HIS3, ARS, CEN*	6
pRS315	*LEU2, ARS, CEN*	6
pRS316	*URA3, ARS, CEN*	6
pRS316-*SNU114*	*URA3, ARS, CEN,*	6
pRS313-*SNU114*	*HIS3, ARS, CEN*, Derivative of pRS313, *SNU114* ORF from pRS316-*SNU114* cloned into SacI and XhoI sites	This work
pRS313-*snu114-777DTLP-As*	Derivative of pRS313-*SNU114*	This work
pRS313-*snu114-814/6/8-As*	Derivative of pRS313-*SNU114*	This work
pRS313-*snu114-T778A*	Derivative of pRS313-*SNU114*	This work
pRS313-*snu114-T778D*	Derivative of pRS313-*SNU114*	This work
pRS315-*SNU114*	*LEU2, ARS, CEN*	
pRS315-*snu114-777DTLP-As*	Derivative of pRS315-*SNU114*	This work
pRS315-*snu114-814/6/8-As*	Derivative of pRS315-*SNU114*	This work
pRS315-*snu114- 60*	Derivative of pRS315-*SNU114*	This work

Supplementary Table T3. Source and description of plasmids used in this study.

References

1. Brenner, T. J. & Guthrie, C. Genetic analysis reveals a role for the C terminus of the Saccharomyces cerevisiae GTPase Snu114 during spliceosome activation. *Genetics* **170,** 1063–1080 (2005).
2. Gautam, A., Grainger, R. J., Vilardell, J., Barrass, J. D. & Beggs, J. D. Cwc21p promotes the second step conformation of the spliceosome and modulates 3' splice site selection. *Nucleic Acids Res.* **43,** 3309–3317 (2015).
3. Nguyen, T. H. D. *et al.* Cryo-EM structure of the yeast U4/U6.U5 tri-snRNP at 3.7 Å resolution. *Nature* **530,** 298–302 (2016).
4. Galej, W. P. *et al.* Cryo-EM structure of the spliceosome immediately after branching. *Nature* **537,** 197–201 (2016).
5. Fromont-Racine, M., Rain, J. C. & Legrain, P. Toward a functional analysis of the yeast genome through exhaustive two-hybrid screens. *Nat. Genet.* **16,** 277–282 (1997).
6. Sikorski, R. S. & Hieter, P. A system of shuttle vectors and yeast host strains designed for efficient manipulation of DNA in Saccharomyces cerevisiae. *Genetics* **122,** 19–27 (1989).
